# Anti-TNF-alpha-induced lupus

**DOI:** 10.1186/s13075-019-2028-2

**Published:** 2019-11-12

**Authors:** Michel De Bandt

**Affiliations:** Unit of Rheumatology, University Hospital Fort-de-France, Centre Hospitalier de Martinique (CHUM), Route de Chateauboeuf, 97200 Fort-de-France, French West Indies France

The publication in March 2005 in *Arthritis Research and Therapy* of the article entitled “Systemic lupus erythematosus induced by anti-tumour necrosis factor alpha therapy: a French national survey” [1] is the result of a long team effort.

At that time, the very young collaborative working group of the CRI (Club Rhumatisme et Inflammation, http://cri-net.com), which brought together the French rheumatology and internal medicine units interested in autoimmune and inflammatory diseases, was in its infancy. Our attention having been drawn 2 years earlier by three cases of anti-TNF-induced lupus, which were in addition to a few rare observations in the literature, we had decided to conduct and complete a French national survey. This was conducted with 866 rheumatology and internal medicine practitioners from all French hospital centers prescribing anti-TNF in rheumatic diseases.

All were registered on the website of the “Club Rhumatismes et Inflammation” and were contacted by email to obtain the files of patients with TNF-induced systemic lupus erythematosus (only infliximab and etanercept, because at that time adalimumab had just been launched in France). Twenty-two cases were collected, revealing two aspects of these manifestations, a rather limited form (association of skin manifestations and anti-DNA) and a more complete form of drug-induced lupus with visceral disorders (CNS, serositis, myositis, arthritis...). The overall trend is in favor of discontinuing anti-TNF drugs (with or without corticosteroids). With the help of Schering-Plough and Wyeth laboratories, the number of patients treated in France was known and a rough incidence calculation was estimated at 0.19% for infliximab and 0.18% for etanercept. While stopping the anti-TNF was beneficial, the reintroduction of another anti-TNF is most often without consequence.

The work had been presented and accepted quickly in a young, newly created journal that was said to be easier to get papers through than in the then giant American journal...but this young journal had understood the importance of the work. The paper had come out without making much noise, but over the years and multiple quotations we had finally understood its importance.... Very quickly, observations of adalimumab-induced lupus were reported, but all anti-TNFs were concerned (class effect also affecting biosimilars), then large published series [2–4]. These are mainly patients with RA, but SPA and MICI are also affected. The mechanisms underlying the genesis of these syndromes remain largely unknown [5].

The development of lupus autoimmunity induced by anti-TNF is a phenomenon already identified in phase II and III studies [6]. Thus, there is a strong biological autoimmunity induced by anti-TNF in systematic monitoring [7–11] of inflammatory rheumatism (with 20 to 60% ANA and 15 to 20% anti-DNA, 15 to 20% anti-Histones). ANAs without specificity have no clinical value. Anti-DNAs are most often of IgM isotype in ELISA and of low affinity. High-affinity antibodies (pathogens in lupus) are associated by IgG isotype and detected by IF or radioimmunological (Farr) tests. However, high rates do not prejudge the appearance of clinical signs and their systematic monitoring is unnecessary. It is noted that there is little induction of other autoantibodies (ACL, anti-tissue, anti-ENA) and in particular less anti-Histones than in lupus induced by hydralazine, procainamide, or beta blockers.

At the clinical level, there are no precise diagnostic criteria for drug-induced lupus and certain sine qua non conditions are retained: the absence of signs of the lupus line in the past, appearance of signs after prolonged exposure to the drug, at least one clinical sign of lupus, and especially disappearance of the signs with the cessation of exposure. The pictures observed often include the skin, joint, muscle, and seritis. Visceral (renal, neurological, etc.) disorders are rarer. The cutaneous form (skin damage in a context of autoimmunity) seems to be the most frequent.

There are traditionally several main groups of lupus-inducing drugs: antiarhythmics (procainamide...), antihypertensives (beta blockers...), antibiotics (minocycline...), anti-convulsive drugs (carbamazepine...), and a recent group represented by immunomodulators (anti-TNF...). About 70 molecules were incriminated.

A recent analysis [12, 13] of the WHO database (VigiBase), which analyzes 8163 “individual case safety report” induced by 118 molecules and collected between 1967 and 2018, shows firstly that in fact about 40 additional molecules cause induced lupus (with a satisfactory statistical confidence level) and secondly that the share of each molecule in the genesis of these diseases has changed considerably over the past 25 years. Indeed, the “weight of each molecule” depends on the importance of its prescription, and some products are no longer used (procainamide, phenytoin, hydralazine...) while the prescription of some others increases considerably. This is the case with anti-TNF agents, which to date represent the first group of lupus-generating molecules induced according to the WHO database.

The pathophysiology of induced lupus is poorly understood, and interactions between drugs (procainamide, hydralazine...) and DNA or Histones leading to a change in immunogenicity (hapten effect) are reported. The frequency of single-stranded anti-DNAs and anti-Histone agents observed in these patients would be consistent with this.

In lupus induced by anti-TNF drugs, it seems different, several hypotheses are proposed without any evidence being provided: (1) an imbalance between interferon-alpha and TNF-alpha, the latter controls the production of IFN first by inhibiting the generation of plasmacytoid dendritic cells (pDCs), a major producer of IFN-alphabeta, from CD34+ hematopoietic progenitors. TNF inhibits IFN-alpha release by immature pDCs exposed to viruses. Neutralization of endogenous TNF sustains IFN-alpha secretion by pDCs [14]. (2) Another hypothesis could be an increase in apoptotic particles and antigens from apoptotic cells. It has been shown that RA patients had no circulating nucleosomes at the steady state and some of them had significantly higher levels of plasma nucleosomes after receiving infliximab [15]. The accumulation of nucleosomes could possibly enhance the development of autoantibodies in subjects with appropriate genetic backgrounds. (3) Another hypothesis [16] is that the suppression of the T-helper type 1 response by TNF blockers could favor a T-helper type 2 response leading to SLE. (4) A final hypothesis [17] is the role of bacterial infections. They are increased with TNF blockers and are also powerful stimulants leading to polyclonal B lymphocyte activation and autoantibody production. Some cases of positive anti-DNA following infection after etanercept have been reported. Interestingly, the titer returned to normal values after antibiotic treatment.

The take home messages are in Table 1.

**Table 1:** Take home messages

- Know that anti-TNF drugs are the first cause of drug-induced lupus. - Do a reference assay of ANA and anti-DNA before starting an anti-TNF. - Do not monitor these rates in the absence of clinical signs. - The presence of ANA before treatment does not contraindicate treatment. - Evoke induced lupus in front of a compatible chronology plus clinical signs and autoantibodies. - Do not diagnose induced lupus on isolated skin damage in an autoimmune context: a “simple” rash or arthralgia with anti-DNA is not sufficient for diagnosis. - Always think about looking for visceral signs. - Stop the product in case of induced lupus and do not take another anti-TNF agent but change class.	

## Data Availability

Not applicable.

## References

[1] De Bandt M, Sibilia J, Le Loët X, Prouzeau S, Fautrel B, Marcelli C, Boucquillard E, Siame JL, Mariette X, the Club Rhumatismes et Inflammation (2005). Systemic lupus erythematosus induced by anti-tumour necrosis factor alpha therapy: a French national survey. Arthritis Res Ther.

[2] Subramanian S, Yajnik V, Sands BE, Cullen G, Korzenik JR (2011). Characterization of patients with infliximab-induced lupus erythematosus and outcomes after retreatment with a second anti-TNF agent. Inflamm Bowel Dis..

[3] Ramos-Casals M, Brito-Zerón P, Muñoz S, Soria N, Galiana D, Bertolaccini L, Cuadrado MJ, Khamashta MA (2007). Autoimmune diseases induced by TNF-targeted therapies: analysis of 233 cases. Medicine (Baltimore)..

[4] Williams VL, Cohen PR (2011). TNF alpha antagonist-induced lupus-like syndrome: report and review of the literature with implications for treatment with alternative TNF alpha antagonists. Int J Dermatol..

[5] He Y, Sawalha AH (2018). Drug-induced lupus erythematosus: an update on drugs and mechanisms. Curr Opin Rheumatol..

[6] Pisetski D (2000). Tumor necrosis factor alpha blockers and the induction of anti-DNA antibodies. Arthritis Rheum..

[7] Charles PJ, Smeenk RJT, De Jong J, Feldmann M, Maini RN (2000). Assessment of antibodies to double-stranded DNA induced in rheumatoid arthritis patients following treatment with infliximab, a monoclonal antibody to tumor necrosis factor α: findings in open-label and randomized placebo-controlled trials. Arthritis Rheum.

[8] Atzeni F, Ardizzone S, Sarzi-Puttini P, Colombo E, Maconi G, De Portu S, Carrabba M, Bianchi Porro G (2005). Autoantibody profile during short-term infliximab treatment for Crohn’s disease: a prospective cohort study. Aliment Pharmacol Ther.

[9] Vermeire S, Noman M, Van Assche G, Baert F, Van Steen K, Esters N, Joossens S, Bossuyt X, Rutgeerts P (2003). Autoimmunity associated with anti-tumor necrosis factor alpha treatment in Crohn's disease: a prospective cohort study. Gastroenterology.

[10] Allanore Y, Sellam J, Batteux F, Job Deslandre C, Weill B, Kahan A (2004). Induction of autoantibodies in refractory rheumatoid arthritis treated by infliximab. Clin Exp Rheumatol.

[11] De Rycke L, Kruithof E, Van Damme N, IEA H, Van den Bossche N, Van den Bosch F, Veys EM, De Keyser F (2003). Antinuclear antibodies following infliximab treatment in patients with rheumatoid arthritis or spondylarthropathy. Arthritis Rheum.

[12] Arnaud L, Mertz P, Gavand P-E, Martin T, Chasset F, Tebacher-Alt M, Lambert A, Muller C, Sibilia J, Lebrun-Vignes B, El Salem J (2019). Drug-induced systemic lupus: revisiting the ever-changing spectrum of the disease using the WHO pharmacovigilance database. Ann Rheum Dis.

[13] Rubin RL (2019). Evolving and expanding scope of lupus-inducing drugs. Ann Rheum Dis.

[14] Palucka AK, Blanck JP, Bennett L, Pascual V, Banchereau J (2005). Cross-regulation of TNF and IFN-alpha in autoimmune diseases. Proc Natl Acad Sci U S A.

[15] D’Auria F, Rovere-Querini P, Giazzon M, Ajello P, Baldissera E, Manfredi A, Sabbadini M (2004). Accumulation of plasma nucleosomes upon treatment with anti-tumour necrosis factor-alpha antibodies. J Intern Med.

[16] Via CS, Shustov A, Rus V, Lang T, Nguyen P, Finkelman FD (2001). In vivo neutralization of TNF-alpha promotes humoral autoimmunity by preventing the induction of CTL. J Immunol.

[17] Ferraccioli G, Mecchia F, Di Poi E, Fabris M (2002). Anticardiolipin antibodies in rheumatoid patients treated with etanercept or conventional combination therapy: direct and indirect evidence for a possible association with infections. Ann Rheum Dis.

